# On designing a new cumulative sum Wilcoxon signed rank chart for monitoring process location

**DOI:** 10.1371/journal.pone.0195762

**Published:** 2018-04-17

**Authors:** Muhammad Abid, Hafiz Zafar Nazir, Muhammad Tahir, Muhammad Riaz

**Affiliations:** 1 Department of Statistics, Government College University, Faisalabad, Pakistan; 2 Department of Statistics, University of Sargodha, Sargodha, Pakistan; 3 Department of Mathematics and Statistics, King Fahad University of Petroleum and Minerals, Dhahran, Saudi Arabia; Universita degli Studi del Piemonte Orientale Amedeo Avogadro, ITALY

## Abstract

In this paper, ranked set sampling is used for developing a non-parametric location chart which is developed on the basis of Wilcoxon signed rank statistic. The average run length and some other characteristics of run length are used as the measures to assess the performance of the proposed scheme. Some selective distributions including Laplace (or double exponential), logistic, normal, contaminated normal and student’s *t*-distributions are considered to examine the performance of the proposed Wilcoxon signed rank control chart. It has been observed that the proposed scheme shows superior shift detection ability than some of the competing counterpart schemes covered in this study. Moreover, the proposed control chart is also implemented and illustrated with a real data set.

## 1. Introduction

Statistical process control (SPC) is a combination of well-known statistical methods and problem-solving methods (such as cause and effect diagram, check sheet, Pareto chart, control chart etc.) that are used to monitor the process (cf. Montgomery [1]). The non-parametric control chart is an emerging area of development in the theory of SPC. Its main advantage is that it does not require any knowledge about the underlying distribution of the variables (cf. Chakraborti et al. [2]).

Different sampling schemes have been used in control charting strategies due to the fact that these play a substantial role in the performance of control charting structures. From one of these sampling schemes, ranked set sampling (RSS) scheme is becoming an important statistical toolkit and it is highly applied in SPC. McIntyre [3] proposed RSS scheme for collecting data in those situations when measurements are damaging or expensive while the ranking of the observation is relatively easy. In RSS scheme first, we select a sample of size *n*^2^ units from the target population and then partition the sample into *n* sets each of size *n*. After that rank the units within each set and then select the smallest ranked unit from the first set, the second smallest ranked unit from the second set, and so on, until the largest ranked unit is selected from the last set as shown in the following demonstration:

$$\begin{array}{ccc} \mathrm{step}\;1 & \mathrm{step}\;2 & \mathrm{step}\;3\\ X_{1,,1}\;X_{2,1}\;\ldots \;X_{n,1} & X_{\left(1\right)1}\;X_{\left(2\right)1}\;\ldots \;X_{\left(n\right)1} & X_{\left(1\right)1}\\ X_{1,,2}\;X_{2,2}\ldots X_{n,2}\;\Leftrightarrow & X_{\left(1\right)2}\;X_{\left(2\right)2}\;\ldots \;X_{\left(n\right)2}\;\Leftrightarrow & X_{\left(2\right)2}\\ \vdots \;\vdots \;\vdots \;\vdots & \vdots \;\vdots \;\vdots \;\vdots & \vdots \\ X_{1,,n}\;X_{2,n}\;\ldots \;X_{n,n} & X_{\left(1\right)n}\;X_{\left(2\right)n}\;\ldots \;X_{\left(n\right)n} & X_{\left(n\right)n} \end{array}$$

These steps are repeated *m* times (cycles) to produce a balanced RSS of size *r* = *nm*. Now a days, RSS has become a useful statistical technique and has been used widely in statistical quality control. Initially, Salazar and Sinha [4] developed control charts by using RSS and median RSS schemes. Muttlak and Al-Sabah [5] are also developed control charts using different RSS schemes. Abujiya and Muttlak [6] developed a control chart on the basis of different double RSS (DRSS) schemes. Al-Omari and Haq [7] also proposed control charts using the DRSS scheme. Abujiya et al. [8] improved the performance of the exponentially weighted moving average (EWMA) control charts by using various forms of RSS schemes. Mehmood et al. [9] introduced the control charts using different RSS schemes to examine the process mean. Abujiya et al. [10] also enhanced cumulative sum (CUSUM) control charts for studying the process dispersion under various RSS schemes. Haq et al. [11] introduced a new synthetic control chart for evaluating process mean and process dispersion by utilizing the various forms of RSS. Haq et al. [12] has also recommended a new Maximum EWMA (Max EWMA) control chart by using the ordered DRSS (ODRSS) and ordered imperfect DRSS (OIDRSS) schemes. The procedure of DRSS and ODRSS schemes are as follows: If we again apply the RSS scheme on *n* ranked set samples, then we will get a double ranked set sample of size *n*. This sampling procedure is called DRSS. If we arrange this double ranked sample in an increasing order of magnitude, we get an ordered double ranked set sample of size *n*, and this scheme is named ODRSS (cf. Haq et al., [12]). If we are faced errors in the ranking procedure of ODRSS, then it is called imperfect double ranked set sampling (OIDRSS) (cf. Haq et al., [12]). Also, Haq et al. [13] developed a new synthetic EWMA and synthetic CUSUM control chart using various forms of RSS schemes for monitoring the process mean.

Recently, some researchers have paid much attention to developing a control chart, based on the RSS method by using a non-parametric approach. Abid et al. [14] developed a non-parametric CUSUM control chart, based on sign test, by using RSS technique. Tapang et al. [15] proposed three non-parametric control charts based on RSS method. A non-parametric EWMA control charts based on sign test and Wilcoxon signed-rank statistic using RSS has also been presented by Abid et al. [16, 17]. In this study, we used the RSS method to develop a non-parametric CUSUM Wilcoxon signed rank control chart to monitor the process target.

The rest of the paper is structured as follows: In Section 2, the concept of the CUSUM signed rank chart by using SRS scheme and the construction of this chart in the RSS scheme is reported. Section 3, consists of the performance evaluation and comparisons of the proposed chart. A real-life use of the suggested scheme is illustrated in Section 4. While a brief conclusion and recommendation are given in Section 5.

## 2. CUSUM signed rank chart using SRS

Bakir and Reynolds [18] proposed the non-parametric CUSUM signed rank chart by utilizing SRS scheme, in future, named as CUSUM-SR chart. According to Bakir and Reynolds [18] the designed structure of the Wilcoxon signed rank (SR) statistic is illustrated below: Let *θ*_0_ be the mean value of the quality characteristic *X*_*ph*_ and it is of the known median, *θ*, that is being tested. Let $R_{ph}^{+}$ be the within-group absolute rank of deviations from the target value *θ*_0_. Mathematically, $R_{ph}^{+}=|X_{ph}-\theta_{0}|,\;h=1,2,\ldots ,n.$

Then, the test statistic of SR under SRS is as follows:

$${SR}_{{SRS}_{p}}=\sum_{h=1}^{n}\;sign\;(X_{ph}-\theta_{0})\;R_{ph}^{+}$$ (1)

where *p* = 1,2,3,… and *h* = 1,2,…,*n* and

$$sign\;(X_{ph}-\theta_{0})=\left\{ \begin{array}{c} 1\;\mathrm{if}\;(X_{ph}-\theta_{0})>0\\ 0\;\mathrm{if}\;(X_{ph}-\theta_{0})=0\\ -1\;\mathrm{if}\;(X_{ph}-\theta_{0})<0 \end{array}\right..$$

According to Gibbons and Chakraborti [19] the SR test statistic is linearly related to another SR test statistic $T_{n}^{+}$ through the relationship:

$${SR}_{SRS}=2T_{n}^{+}-n(n+1)/2$$ (2)

The mean and variance of $T_{n}^{+}$ are $E(T_{n}^{+})=n(n+1)/4$ and $Var(T_{n}^{+})=n(n+1)(2n+1)/24$, respectively (cf. Gibbons and Chakraborti [19]). Using the relationship in Eq (2), we get the mean and variance of *SR*_*SRS*_ as *E*(*SR*_*SRS*_) = 0 and $Var({SR}_{SRS})=\sigma_{{SR}_{SRS}}=n(n+1)(2n+1)/6$, respectively.

The two plotting and monitoring statistics $C_{p}^{+}$ and $C_{p}^{-}$ of the CUSUM-SR scheme using Eq (1) are defined as:

$$\begin{array}{c} C_{p}^{+}=max\;[0,(SR_{SRS_{p}})-K+C_{p-1}^{+}]\\ C_{p}^{-}=max\;[0,-(SR_{SRS_{p}})-K+C_{p-1}^{-}] \end{array}\},\mathrm{where}\;p=1,2\ldots$$ (3)

where ${SR}_{{SRS}_{p}}$ is the SR statistic at time *p* and *K* is the reference value of the CUSUM-SR scheme (cf. Montgomery [1]). Initial values of $C_{p}^{+}$ and $C_{p}^{-}$ are set as zero i.e., $C_{p}^{+}=0$ and $C_{p}^{-}=0.$ After that, $C_{p}^{+}$ and $C_{p}^{-}$ are plotted against the control limit *H*. If any $C_{p}^{+}>H$ or $C_{p}^{-}>H$, the process is declared to be out of control, if not, it is in control. So, the CUSUM-SR chart has two parameters *K* and *H*. These two parameters are defined as (cf. Montgomery [1]).

$$K=k{\hat{\sigma }}_{SR_{SRS}},\;H=h{\hat{\sigma }}_{SR_{SRS}}$$ (4)

where ${\hat{\sigma }}_{{SR}_{SRS}}=\frac{\sigma_{{SR}_{SRS}}}{\sqrt{n}}$.

### 2.1. Proposed CUSUM signed rank chart using RSS

Let a sample of size *n* is obtained from *X*_(*ph*)*i*_ using RSS to examine the process location, *θ*_0_. Let $R_{(ph)i}^{+}$ be the within-group absolute rank of deviations from the target value *θ*_0_. Mathematically, $R_{(ph)i}^{+}=|X_{(ph)i}-\theta_{0}|.$ Let, *r* = *nm*,*m* > 1 gives a RSS and *m* be the number of cycles to be repeat.

The test statistic of SR under RSS is as follows (cf. Kim and Kim [20])

$${SR}_{{RSS}_{p}}=\sum_{h=1}^{n}\sum_{i=1}^{m}\;sign\;(X_{(ph)i}-\theta_{0})\;R_{(ph)i}^{+},$$ (5)

where, *p* = 1,2,3,…, *h* = 1,2,…,*n* and *i* = 1,2,…*m* and

$$sign\;(X_{(ph)i}-\theta_{0})=\left\{ \begin{array}{c} 1\;\mathrm{if}\;(X_{(ph)i}-\theta_{0})>0\\ 0\;\mathrm{if}\;(X_{(ph)i}-\theta_{0})=0\\ -1\;\mathrm{if}\;(X_{(ph)i}-\theta_{0})<0 \end{array}\right..$$

The test statistic of SR using RSS is also linearly linked to another test statistic of SR i.e., $W_{RSS}^{+}$ through the relationship:

$${SR}_{RSS}=2W_{RSS}^{+}-nm(m+1)/2$$ (6)

The expected value and variance of the statistic $W_{RSS}^{+}$ are as follow: $E(W_{RSS}^{+})=nm(m+1)/4$ and $Var(W_{RSS}^{+})=\left(nm\;(m+1)(2m+1\right)/24{)\delta }_{0}^{2}$, respectively (cf. Kim and Kim [20]). So, the mean and variance of *SR*_*RSS*_, through the relationship mentioned in Eq (6), are given as: *E*(*SR*_*RSS*_) = 0 and $Var({SR}_{RSS})=\sigma_{{SR}_{RSS}}=(nm(m+1)(2m+1)/6)\delta_{0}^{2}$, respectively. The value of $\delta_{0}^{2}$ can be defined as:

δ02=1−(4n)∑h=1n(Fh(0)−12)2. (7)

where the values of *F*_*h*_(0), *h* = 1,2,…,*n*, are obtained byestimating the incomplete beta integral.

$$F_{h}(0)=\frac{r!}{(h-1)!(r-h)!}\int_{-\propto }^{0}F{\left(u\right)}^{h-1}{\left(1-F(u)\right)}^{r-h}f(u)du$$ (8)

The values of $\delta_{0}^{2}$, from *n* = 2,3,…, 10, are reported in Table 1 which is a vital part to get the enhancement of RSS over SRS (cf. Hettmansperger [21]).

**Table 1:** Values of *F*_*h*_(0) and $\delta_{0}^{2}$.

*n*	2	3	4	5	6	7	8	9	10
1	0.750	0.875	0.938	0.969	0.984	0.992	0.996	0.998	0.999
2	0.250	0.500	0.688	0.813	0.891	0.938	0.965	0.981	0.989
3		0.125	0.313	0.500	0.656	0.773	0.856	0.910	0.945
4			0.063	0.188	0.344	0.500	0.637	0.746	0.828
5				0.031	0.109	0.227	0.363	0.500	0.623
6					0.016	0.063	0.145	0.254	0.377
7						0.008	0.035	0.090	0.172
8							0.004	0.020	0.055
9								0.002	0.011
10									0.001
$\delta_{0}^{2}$	0.750	0.625	0.547	0.490	0.451	0.416	0.393	0.371	0.352

So, the two plotting and monitoring statistics i.e., $C_{p}^{+}$ and $C_{p}^{-}$ of the proposed non-parametric CUSUM signed rank control chart, later on named as, RCUSUM-SR chart based on Eq (5) can be written as:

$$\begin{array}{c} C_{p}^{+}=max[0,{(SR}_{{RSS}_{p}})-K+C_{p-1}^{+}]\\ C_{p}^{-}=max[0,{-(SR}_{{RSS}_{p}})-K+C_{p-1}^{-}] \end{array}\},\;\mathrm{where}\;p=1,2\ldots$$ (9)

where ${SR}_{{RSS}_{p}}$ is the SR statistic of the proposed chart at time *p* and *K* is the reference value of the suggested chart (cf. Montgomery [1]). The initial value of the two plotting statistics, for the proposed scheme, is taken equal to zero i.e., $C_{p}^{+}=0$ and $C_{p}^{-}=0.$ Next, the two plotting and monitoring statistics, given in Eq (9), are plotted against the control limit *H*. As long as the values of $C_{p}^{+}$ and $C_{p}^{-}$ are plotted inside of the control limit i.e., *H*, then the process is assumed to be in control, if not, it is out of control. The *K* and *H* are two design parameters of the proposed chart and these parameters are selected in a way that they satisfy average run length (*ARL*). The two parameters of the proposed chart are defined as (cf. Montgomery [1]).

$$K=k{\hat{\sigma }}_{{SR}_{RSS}},\;H=h{\hat{\sigma }}_{{SR}_{RSS}}$$ (10)

where ${\hat{\sigma }}_{{SR}_{RSS}}=\frac{\sigma_{{SR}_{RSS}}}{\sqrt{r}}.$

We evaluate that combinations of (*K*,*H*) by considering different values of *n* and *m* under which the proposed chart has its *ARL*_0_ close to 370 and 500 (i.e. *ARL*_0_ ≅ 370 and 500). The values of (*K*,*H*) of the proposed RCUSUM-SR chart are given in Table 2. From Table 2, we found that increase in the values of *n* and *m* may also cause increase in the value of *K* and vice versa. There is also found an upward and downward pattern in the value of *H* by increasing the values of *n* and *m* (cf. Table 2). Table 2 plays a vital role for implementing the proposed chart in practice.

**Table 2:** Design parameters values for various choices of (*n*,*m*) for the proposed chart.

*Nominal ARL*_0_ ≈ 370			*Nominal ARL*_0_ ≈ 500		
*n*	*m*	(*K*,*H*)	*n*	*m*	(*K*,*H*)
5	1	(2, 11.10)	5	1	(2, 11.25)
6		(2, 13.80)	6		(2, 14.52)
8		(4, 10.35)	8		(4, 11.25)
10		(4, 14.10)	10		(4, 15.10)
5	3	(3, 10.80)	5	3	(3, 11.50)
6		(4, 10.70)	6		(4, 11.30)
8		(6, 10.90)	8		(6, 11.50)
10		(6, 15.30)	10		(6, 16.23)
5	5	(4, 9.00)	5	5	(4, 9.50)
6		(4, 12.00)	6		(4, 12.52)
8		(6, 12.30)	8		(6, 13.13)
10		(6, 17.30)	10		(6, 18.40)

## 3. Performance assessment of the proposed chart

In this section, we assessed the performance of the proposed RCUSUM-SR control chart for in control and out of control processes. The average run length (*ARL*), which is the mean of run length (*RL*) distribution, is the most significant and commonly used measure to assess the performance of control charts. Some researchers such as Barnard [22], Woodall [23] and Gan [24] have discouraged the only use of *ARL* due to the skewed distribution of run length. Hence, in this study, we also include some other measures of run length such as the standard deviation (*SDRL*) and some percentile points (which includes 5th, 25th, 50th, 75th and 95th) in order to enhance better results.

In this study, we include a variety of distributions such as: (a) the standard normal distribution, *N*(0,1); (b) the Student’s *t*-distribution, *t*(*v*), with degree of freedom *v* = 4 and 8, respectively; (c) the Laplace (or double exponential) distribution, $DE(0,1/\sqrt{2})$; (d) the logistic distribution, $LOG(0,\sqrt{3}/\pi )$; the contaminated normal (*CN*) distribution, a mixture of $N(0,\sigma_{1}^{2})$ and $N(0,\sigma_{2}^{2})$, represented by $(1-\alpha )N(0,\sigma_{1}^{2})+\alpha N(0,\sigma_{2}^{2}),$ where $(1-\alpha )\sigma_{1}^{2}+\alpha \sigma_{2}^{2}=1$ and *α* = 0.05. The main use of *CN* distribution is to evaluate the performance of proposed chart in case of outliers. The distributions considered in this study, having mean value 0 and the value of standard deviation 1, in order to compare the results of all distributions.

In this study, a Monte Carlo (MC) simulation is used to calculate the run length values of the RCUSUM-SR chart and R language is used for this purpose. The simulations steps are given below:

1. We create random subgroup from all the selected distributions using the values of *K*,*H*,*m*,*n* and shift of the size (*δ*) for a given *ARL*_0_.
2. The proposed chart is constructed by using the expression given in Eq (9).
3. The number of samples, at which the plotting statistic of the proposed chart falls outside the control limit *H*, is observed.
4. The steps (i)-(iv) are repeated 50000 to obtain the run lengths.

At the end, we found the average of the values in step (iv) to get the *ARL* value.The in control and out of control performances of the RCSUSM-SR chart is compared with the charts selected in this study. The performance assessment is made on the basis of *ARL*. The proposed chart is compared with the CUSUM-SR chart proposed by Bakir and Reynolds [18], the nonparametric EWMA signed rank chart using RSS developed by Abid et al. [17] hereafter, is named as REWMA-SR control chart, the nonparametric EWMA signed rank chart using SRS constructed by Graham et al. [25], after that, is named as EWMA-SR control chart and the noparametric EWMA sign chart proposed by Graham et al. [26] and is named as EWMA-SN control chart. As the proposed and the existing charts are non-parametric control charts, therefore, their in control run length distribution have to remain similar for all continuous distributions and these results are reported in Table 3 at *ARL*_0_ ≈ 500. It is to be noted that the *ARL*_0_ of the proposed chart and the existing charts seems almost identical for all distributions under consideration in this study (cf. Table 3). The first row of each cell in Tables 3–7 shows the ARL and SDRL values, respectively, whereas the second row shows the values of the 5th, 25th, 50th, 75th and 95th percentiles (in this order).

**Table 3:** In control performance of the proposed and existing control charts for *n* = 10 and *m* = 3.

**RCUSUM – SR chart**	
(*K*,*H*)	**(6, 16.23)**
**For all distributions**	501.74 (494.39) 28, 147, 361, 709, 1506
**CUSUM – SR chart**	
(*K*,*H*)	**(1, 2.40)**
**For all distributions**	498.43 (489.84) 29, 144, 345, 685, 1460
**REWMA – SR chart**	
**(*λ*,*L*)**	**(0.05, 1.925)**
**For all distributions**	498.81 (479.16) 41, 160, 346, 706, 1485
**EWMA – SR chart**	
**(*λ*,*L*)**	**(0.05, 2.610)**
**For all distributions**	500.67 (486.10) 40, 154, 352, 688, 1471
**EWMA – SN chart**	
**(*λ*,*L*)**	**(0.05, 2.612)**
**For all distributions**	501.04 (486.58) 39, 155, 352, 689, 1472

**Table 4:** Out of control performance of the proposed scheme for *n* = 5.

RCUSUM – SR chart							
Dist.	*m*	(*K*,*H*)	*δ*				
			0.5	1.0	1.5	2.0	2.5
***N*(0,1)**	**1**	**(2, 11.2)**	4.94 (2.04) 2, 4, 5, 6, 9	2.38 (0.58) 2, 2, 2, 3, 3	2.02 (0.15) 2, 2, 2, 2, 2	2.00 (0.01) 2, 2, 2, 2, 2	2.00 (0.00) 2, 2, 2, 2, 2
***t*(4)**			3.95 (1.52) 2, 3, 4, 5, 7	2.27 (0.52) 2, 2, 2, 2, 3	2.05 (0.23) 2, 2, 2, 2, 2	2.02 (0.13) 2, 2, 2, 2, 2	2.00 (0.10) 2, 2, 2, 2, 2
***t*(8)**			4.57 (1.82) 3, 3, 5, 6, 8	2.34 (0.56) 2, 2, 2, 3, 3	2.04 (0.19) 2, 2, 2, 2, 2	2.01 (0.07) 2, 2, 2, 2, 2	2.00 (0.03) 2, 2, 2, 2, 2
***Laplace***			2.37 (1.63) 1, 1, 2, 3, 6	1.73 (1.02) 1, 1, 1, 2, 4	1.53 (0.82) 1, 1, 1, 2, 3	1.48 (0.75) 1, 1, 1, 2, 3	1.44 (0.73) 1, 1, 1, 2, 3
***Logistic***			11.22 (6.98) 4, 6, 9, 14, 25	4.04 (1.52) 2, 3, 4, 5, 7	2.68 (0.80) 2, 2, 3, 3, 4	2.22 (0.47) 2, 2, 2, 2, 3	2.07 (0.26) 2, 2, 2, 2, 3
***CN***			4.77 (1.91) 3, 3, 4, 6, 8	2.36 (0.57) 2, 2, 2, 3, 3	2.03 (0.18) 2, 2, 2, 2, 2	2.00 (0.06) 2, 2, 2, 2, 2	2.00 (0.03) 2, 2, 2, 2, 2
***N*(0,1)**	**3**	**(3, 11.5)**	2.14 (0.63) 1, 2, 2, 2, 3	1.12 (0.32) 1, 1, 1, 1, 2	1.00 (0.01) 1, 1, 1, 1, 1	1.00 (0.00) 1, 1, 1, 1, 1	1.00 (0.00) 1, 1, 1, 1, 1
***t*(4)**			1.85 (0.55) 1, 2, 2, 2, 3	1.09 (0.29) 1, 1, 1, 1, 2	1.00 (0.06) 1, 1, 1, 1, 1	1.00 (0.03) 1, 1, 1, 1, 1	1.00 (0.01) 1, 1, 1, 1, 1
***t*(8)**			2.04 (0.59) 1, 2, 2, 2, 3	1.12 (0.32) 1, 1, 1, 1, 2	1.01 (0.09) 1, 1, 1, 1, 1	1.00 (0.01) 1, 1, 1, 1, 1	1.00 (0.00) 1, 1, 1, 1, 1
***Laplace***			4.05 (1.76) 2, 3, 4, 5, 7	1.01 (0.09) 1, 1, 1, 1, 2	1.00 (0.03) 1, 1, 1, 1, 1	1.00 (0.02) 1, 1, 1, 1, 1	1.00 (0.01) 1, 1, 1, 1, 1
***Logistic***			2.44 (0.76) 1, 2, 2, 3, 5	1.87 (0.54) 1, 2, 2, 2, 3	1.31 (0.46) 1, 1, 1, 2, 2	1.06 (0.24) 1, 1, 1, 1, 2	1.00 (0.08) 1, 1, 1, 1, 1
***CN***			2.09 (0.59) 1, 2, 2, 2, 3	1.11 (0.32) 1, 1, 1, 1, 2	1.01 (0.04) 1, 1, 1, 1, 1	1.00 (0.01) 1, 1, 1, 1, 1	1.00 (0.01) 1, 1, 1, 1, 1

**Table 5:** Out of control performance of the proposed scheme for *n* = 10.

RCUSUM – SR chart							
Dist.	*m*	(*K*,*H*)	*δ*				
			0.5	1.0	1.5	2.0	2.5
***N*(0,1)**	**1**	**(4, 15.10)**	1.98 (0.56) 1, 2, 2, 2, 3	1.06 (0.23) 1, 1, 1, 1, 2	1.00 (0.01) 1, 1, 1, 1, 1	1.00 (0.00) 1, 1, 1, 1, 1	1.00 (0.00) 1, 1, 1, 1, 1
***t*(4)**			1.72 (0.53) 1, 1, 2, 2, 2	1.07 (0.26) 1, 1, 1, 1, 2	1.02 (0.13) 1, 1, 1, 1, 1	1.00 (0.08) 1, 1, 1, 1, 1	1.00 (0.06) 1, 1, 1, 1, 1
***t*(8)**			1.88 (0.54) 1, 2, 2, 2, 3	1.07 (0.25) 1, 1, 1, 1, 2	1.00 (0.07) 1, 1, 1, 1, 1	1.00 (0.02) 1, 1, 1, 1, 1	1.00 (0.01) 1, 1, 1, 1, 1
***Laplace***			1.41 (0.71) 1, 2, 2, 2, 3	1.15 (0.40) 1, 1, 1, 1, 2	1.09 (0.31) 1, 1, 1, 1, 2	1.07 (0.26) 1, 1, 1, 1, 2	1.06 (0.26) 1, 1, 1, 1, 2
***Logistic***			3.59 (1.55) 2, 3, 3, 4, 7	1.73 (0.51) 1, 1, 2, 2, 2	1.20 (0.41) 1, 1, 1, 1, 2	1.04 (0.19) 1, 1, 1, 1, 1	1.01 (0.08) 1, 1, 1, 1, 1
***CN***			1.93 (0.55) 1, 2, 2, 2, 3	1.06 (0.24) 1, 1, 1, 1, 2	1.01 (0.06) 1, 1, 1, 1, 1	1.00 (0.02) 1, 1, 1, 1, 1	1.00 (0.01) 1, 1, 1, 1, 1
***N*(0,1)**	**3**	**(6, 16.23)**	1.01 (0.13) 1, 1, 1, 1, 1	1.00 (0.00) 1, 1, 1, 1, 1	1.00 (0.00) 1, 1, 1, 1, 1	1.00 (0.00) 1, 1, 1, 1, 1	1.00 (0.00) 1, 1, 1, 1, 1
***t*(4)**			1.00 (0.03) 1, 1, 1, 1, 1	1.00 (0.00) 1, 1, 1, 1, 1	1.00 (0.00) 1, 1, 1, 1, 1	1.00 (0.00) 1, 1, 1, 1, 1	1.00 (0.00) 1, 1, 1, 1, 1
***t*(8)**			1.01 (0.08) 1, 1, 1, 1, 1	1.00 (0.00) 1, 1, 1, 1, 1	1.00 (0.00) 1, 1, 1, 1, 1	1.00 (0.00) 1, 1, 1, 1, 1	1.00 (0.00) 1, 1, 1, 1, 1
***Laplace***			1.00 (0.03) 1, 1, 1, 1, 1	1.00 (0.00) 1, 1, 1, 1, 1	1.00 (0.00) 1, 1, 1, 1, 1	1.00 (0.00) 1, 1, 1, 1, 1	1.00 (0.00) 1, 1, 1, 1, 1
***Logistic***			1.61 (0.57) 1, 1, 2, 2, 2	1.00 (0.02) 1, 1, 1, 1, 1	1.00 (0.00) 1, 1, 1, 1, 1	1.00 (0.00) 1, 1, 1, 1, 1	1.00 (0.00) 1, 1, 1, 1, 1
***CN***			1.06 (0.24) 1, 1, 1, 1, 2	1.00 (0.00) 1, 1, 1, 1, 1	1.00 (0.00) 1, 1, 1, 1, 1	1.00 (0.00) 1, 1, 1, 1, 1	1.00 (0.00) 1, 1, 1, 1, 1

**Table 6:** Out of control performance of the CUSUM – SR chart for *n* = 10.

CUSUM – SR chart						
Dist.	(*K*,*H*)	*δ*				
		0.5	1.0	1.5	2.0	2.5
***N*(0,1)**	**(1, 2.42)**	6.17 (3.64) 2, 4, 5, 8, 13	2.44 (0.64) 2, 2, 2, 3, 4	2.02 (0.14) 2, 2, 2, 2, 2	2.00 (0.02) 2, 2, 2, 2, 2	2.00 (0.00) 2, 2, 2, 2, 2
***t*(4)**		4.59 (2.33) 2, 3, 4, 6, 9	2.29 (0.52) 2, 2, 2, 3, 3	2.03 (0.18) 2, 2, 2, 2, 2	2.00 (0.06) 2, 2, 2, 2, 2	2.00 (0.03) 2, 2, 2, 2, 2
***t*(8)**		5.53 (3.09) 2, 4, 5, 7, 12	2.38 (0.59) 2, 2, 2, 3, 3	2.02 (0.17) 2, 2, 2, 2, 2	2.00 (0.03) 2, 2, 2, 2, 2	2.00 (0.01) 2, 2, 2, 2, 2
***Laplace***		8.13 (5.55) 3, 4, 7, 10, 19	3.06 (1.11) 2, 2, 3, 4, 5	2.28 (0.51) 2, 2, 2, 3, 3	2.06 (0.25) 2, 2, 2, 2, 3	2.01 (0.11) 2, 2, 2, 2, 2
***Logistic***		20.57 (17.60) 4, 8, 15, 28, 56	4.68 (2.36) 2, 3, 4, 6, 9	2.78 (0.88) 2, 2, 3, 3, 4	2.25 (0.48) 2, 2, 2, 2, 3	2.06 (0.25) 2, 2, 2, 2, 3
***CN***		5.82 (3.32) 2, 3, 5, 7, 12	2.40 (0.60) 2, 2, 2, 3, 3	2.03 (0.16) 2, 2, 2, 2, 2	2.00 (0.02) 2, 2, 2, 2, 2	2.00 (0.01) 2, 2, 2, 2, 2

**Table 7:** Out of control performance of the REWMA − SR, the EWMA − SR and EWMA − SN charts for *λ* = 0.05, *m* = 1 and *n* = 10.

**REWMA – SR chart**						
**Dist.**	***L***	***δ***				
		**0.5**	**1.0**	**1.5**	**2.0**	**2.5**
***N*(0,1)**	**1.995**	3.33 (0.59) 3, 3, 3, 4, 4	2.04 (0.20) 2, 2, 2, 2, 2	2.00 (0.00) 2, 2, 2, 2, 2	2.00 (0.00) 2, 2, 2, 2, 2	2.00 (0.00) 2, 2, 2, 2, 2
***t*(4)**		2.49 (0.51) 2, 3, 3, 3, 4	2.03 (0.19) 2, 2, 2, 2, 2	2.00 (0.03) 2, 2, 2, 2, 2	2.00 (0.01) 2, 2, 2, 2, 2	2.00 (0.01) 2, 2, 2, 2, 2
***t*(8)**		3.20 (0.55) 2, 3, 3, 4, 4	2.05 (0.22) 2, 2, 2, 2, 2	2.00 (0.01) 2, 2, 2, 2, 2	2.00 (0.00) 2, 2, 2, 2, 2	2.00 (0.00) 2, 2, 2, 2, 2
***Laplace***		3.79 (0.78) 3, 3, 4, 4, 5	2.41 (0.50) 2, 2, 2, 3, 3	2.04 (0.20) 2, 2, 2, 2, 3	2.00 (0.05) 2, 2, 2, 2, 2	2.00 (0.02) 2, 2, 2, 2, 2
***Logistic***		5.35 (1.29) 4, 4, 5, 6, 8	2.99 (0.50) 2, 3, 3, 3, 4	2.23 (0.43) 2, 2, 2, 2, 3	2.02 (0.14) 2, 2, 2, 2, 2	2.00 (0.03) 2, 2, 2, 2, 2
***CN***		3.27 (0.57) 2, 3, 3, 4, 4	2.05 (0.21) 2, 2, 2, 2, 2	2.00 (0.02) 2, 2, 2, 2, 2	2.00 (0.00) 2, 2, 2, 2, 2	2.00 (0.00) 2, 2, 2, 2, 2
**EWMA – SR chart**						
**Dist.**	***L***	***δ***				
		**0.5**	**1.0**	**1.5**	**2.0**	**2.5**
***N*(0,1)**	**2.610**	7.65 (1.97) 5, 6, 7, 9, 11	4.46 (0.58) 4, 4, 4, 5, 5	4.00 (0.07) 4, 4, 4, 4, 4	4.00 (0.00) 4, 4, 4, 4, 4	4.00 (0.00) 4, 4, 4, 4, 4
***t*(4)**		6.51 (1.47) 5, 5, 6, 7, 9	4.27 (0.47) 4, 4, 4, 5, 5	4.01 (0.11) 4, 4, 4, 4, 4	4.00 (0.02) 4, 4, 4, 4, 4	4.00 (0.01) 4, 4, 4, 4, 4
***t*(8)**		7.21 (1.77) 5, 6, 7, 8, 10	4.39 (0.55) 4, 4, 4, 5, 5	4.01 (0.09) 4, 4, 4, 4, 4	4.00 (0.01) 4, 4, 4, 4, 4	4.00 (0.00) 4, 4, 4, 4, 4
***Laplace***		6.54 (1.51) 5, 5, 6, 7, 9	4.34 (0.52) 4, 4, 4, 5, 5	4.02 (0.13) 4, 4, 4, 4, 4	4.00 (0.02) 4, 4, 4, 4, 4	4.00 (0.00) 4, 4, 4, 4, 4
***Logistic***		7.20 (1.77) 5, 6, 7, 8, 10	4.39 (0.55) 4, 4, 4, 5, 5	4.01 (0.10) 4, 4, 4, 4, 4	4.00 (0.01) 4, 4, 4, 4, 4	4.00 (0.00) 4, 4, 4, 4, 4
***CN***		7.42 (1.87) 5, 6, 7, 8, 11	4.41 (0.56) 4, 4, 4, 5, 5	4.01 (0.08) 4, 4, 4, 4, 4	4.00 (0.01) 4, 4, 4, 4, 4	4.00 (0.00) 4, 4, 4, 4, 4
**EWMA – SR chart**						
**Dist.**	***L***	***δ***				
		**0.5**	**1.0**	**1.5**	**2.0**	**2.5**
***N*(0,1)**	**2.612**	9.01 (2.76) 5, 7, 9, 11, 14	4.78 (0.85) 4, 4, 5, 5, 6	3.65 (0.57) 3, 3, 4, 4, 4	3.15 (0.35) 3, 3, 3, 3, 4	3.01 (0.12) 3, 3, 3, 3, 3
***t*(4)**		6.94 (1.76) 5, 6, 7, 8, 10	4.21 (0.69) 3, 4, 4, 5, 5	3.47 (0.53) 3, 3, 3, 4, 4	3.16 (0.37) 3, 3, 3, 3, 4	3.05 (0.22) 3, 3, 3, 3, 4
***t*(8)**		8.08 (2.31) 5, 6, 8, 9, 12	4.53 (0.77) 3, 4, 4, 5, 6	3.58 (0.56) 3, 3, 4, 4, 4	3.17 (0.38) 3, 3, 3, 3, 4	3.04 (0.19) 3, 3, 3, 3, 3
***Laplace***		6.56 (1.59) 5, 5, 6, 7, 9	4.29 (0.71) 3, 4, 4, 5,5	3.57 (0.55) , 3, 3, 4, 4, 4	3.22 (0.42) 3, 3, 3, 3, 4	3.07 (0.25) 3, 3, 3, 3, 4
***Logistic***		8.00 (2.26) 5, 6, 8, 9, 12	4.53 (0.77) 3, 4, 4, 5, 6	3.59 (0.56) 3, 3, 4, 4, 4	3.18 (0.39) 3, 3, 3, 3, 4	3.04 (0.20) 3, 3, 3, 3, 3
***CN***		8.61 (2.57) 5, 7, 8, 10, 13	4.65 (0.81) 4, 4, 5, 5, 6	3.59 (0.56) 3, 3, 4, 4, 4	3.14 (0.35) 3, 3, 3, 3, 4	3.02 (0.15) 3, 3, 3, 3, 3

The *ARL*_1_ values of the RCUSUM-SR chart for all the selected distributions and *δ* = 0.5(0.5)2.5 (shift in the process), for *n* = 5, 10 and *m* = 1(2)3 are given in Tables 4 and 5.

The main findings from Tables 4 and 5 are as follows:

1. The *ARL*_1_ values of the RCUSUM-SR scheme increase as the value of *n* increases and vice versa (cf. Table 4 vs Table 5).
2. It is found to be increase in the values of *ARL*_1_ when the value of *m* decreases and vice versa (cf. Table 4).
3. The out of control performance of the proposed scheme is relatively better in case of *t*(4) and *t*(8) when *δ* ≤ 0.1, as compared to other distributions selected in this study (cf. Tables 4 and 5).
4. It is also found that the performance of the RCUSUM-SR chart is relatively better in case of CN distribution in comparison to other distributions such as *N*(0,1), $DE(0,1/\sqrt{2})$ and $LOG(0,\sqrt{3}/\pi )$ when *δ* ≤ 0.1 (cf. Tables 4 and 5).
5. The *SDRL* and percentiles values also increases when the values of *m* and *n* decrease and vice versa (cf. Table 4 vs Table 5).

So, in general, we can say that the proposed scheme is more proficient in detecting the small and large shifts in the location parameter.

### 3.1. Comparisons

This section is related to the comparison of the RCUSUM-SR chart with the charts considered in this study. The proposed chart is compared with the CUSUM-SR, the REWMA-SR, the EWMA-SR and the EWMA-SN charts on the basis of *ARL*. We have also regenerated the results of Abid et al. [17] Bakir and Reynolds [18], Graham et al. [25] and Graham et al. [26] and found similar results.

#### 3.1.1. RCUSUM-SR versus CUSUM-SR

The *ARL*_1_ values of the CUSUM-SR scheme are reported in Table 6. From Tables 5 and 6, it is revealed that the *ARL*_1_ performance of the RCUSUM-SR chart is quite better in comparison to CUSUM-SR chart for all the values of *m* and *n* under all the selected distributions. The proposed scheme has smaller *ARL*_1_ values as compared to the CUSUM-SR scheme for all the selected shifts (cf. Table 5 versus Table 6). Hence, the proposed scheme performs more efficiently under all conditions in comparison to CUSUM-SR scheme.

#### 3.1.2. RCUSUM-SR versus EWMA-SN

The results of EWMA-SN scheme are given in Table 7. From Tables 5 and 7, it is observed that the RCUSUM-SR scheme has better *ARL*_1_ performance in comparison to the EWMA-SN scheme for all choices of design parameters under all the selected distributions. Thus, we can say that RCUSUM-SR has a better performance in detecting all the selected shifts than the EWMA-SN chart.

#### 3.1.3. RCUSUM-SR versus EWMA-SR

The *ARL*_1_ values of the EWMA-SR chart are provided in Table 7. Comparing the EWMA-SR chart with the RCUSUM-SR chart, it is noted that RCUSUM-SR chart performs more efficiently than the EWMA-SR scheme for detecting all the selected shifts under all the selected distributions (cf. Table 5 versus Table 7). Hence, the proposed RCUSUM-SR chart shows reasonably improved performance in comparison to the EWMA-SR scheme.

#### 3.1.4. RCUSUM-SR versus REWMA-SR

The *ARL*_1_ results of REWMA-SR scheme are given in Table 7. To compare the proposed scheme with the REWMA-SR, we observed that the RCUSUM-SR scheme shows improved shift detection ability than the REWMA-SR scheme under normal and non-normal distributions (cf.Table 5 versus Table 7). Thus, the proposed chart performs more efficiently as compared to the REWMA-SR scheme.

**Fig 1:**
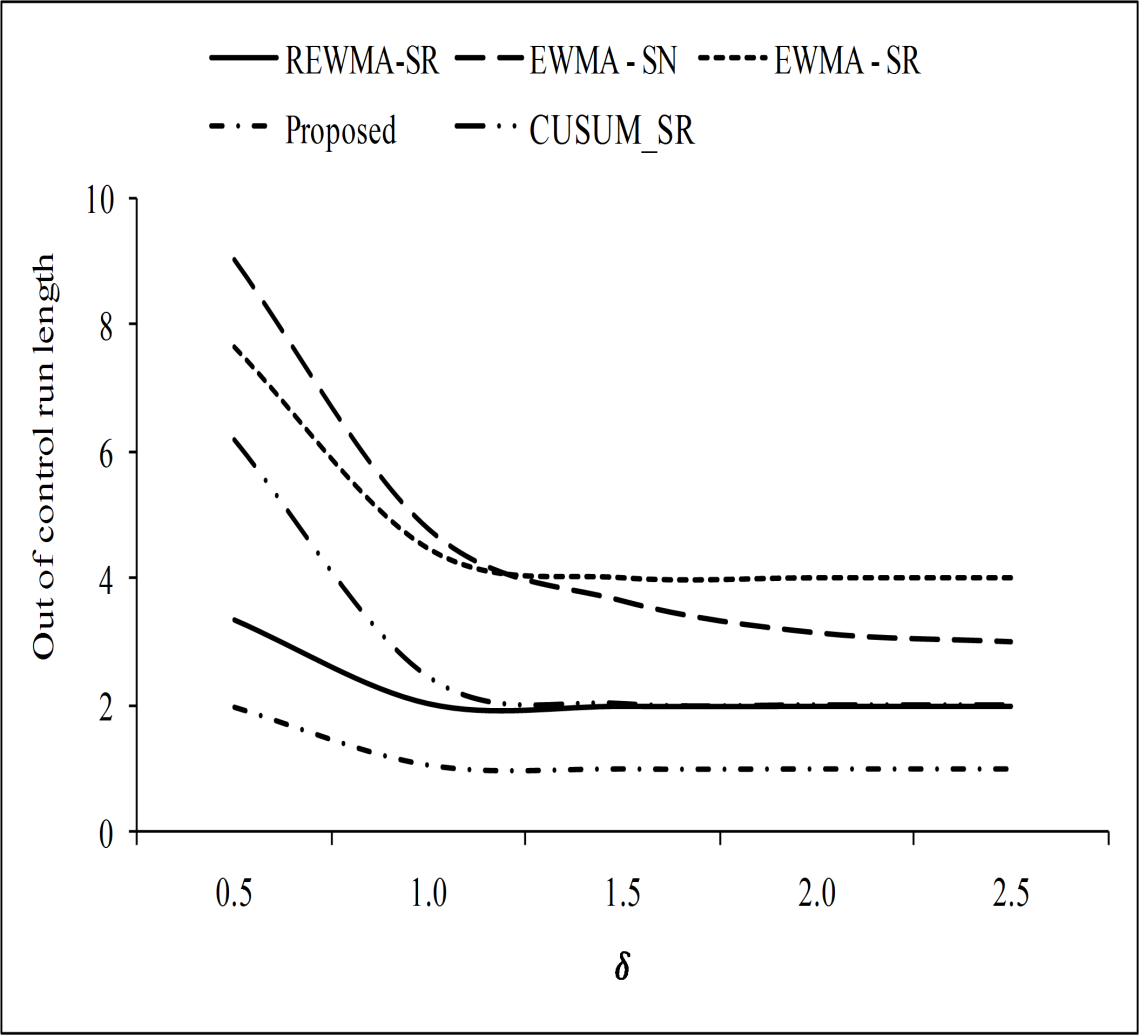
Comparison of the proposed chart with other charts considered in this study at *n* = 10 and *m* = 1.

**Fig 2:**
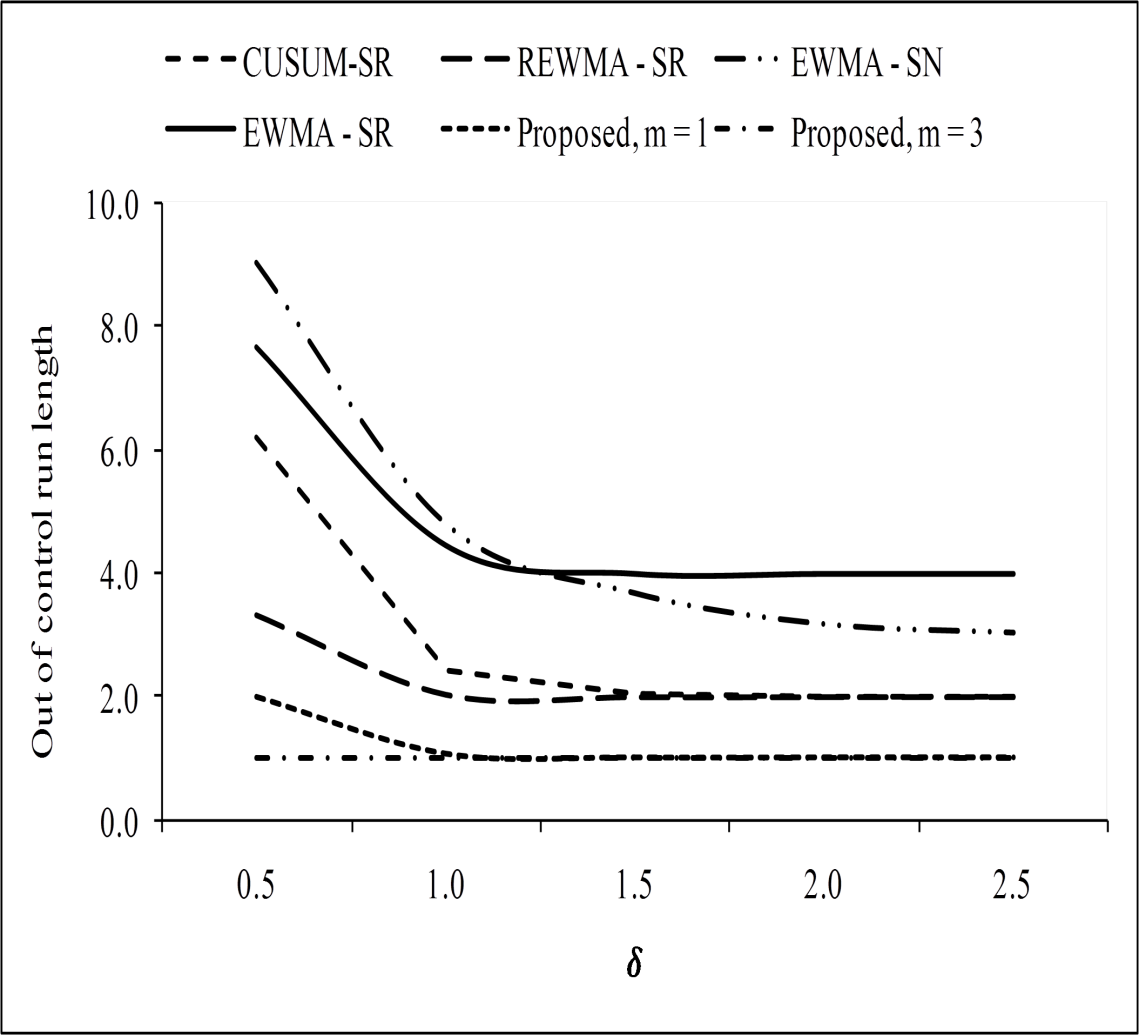
Comparison of the proposed chart with other charts considered in this study at *n* = 10.

To prove the superiority of the proposed chart over other existing charts, we have made some graphical presentation in the form of *ARL*_1_ curve. In Figs 1 and 2, the proposed RCUSUM-SR scheme is compared with the CUSUM-SR, the REWMA-SR, the EWMA-SR and the EWMA-SN charts. From Fig 1, it can be seen that the proposed chart shows better performance as compared with the CUSUM-SR, the REWMA-SR, the EWMA-SR and the EWMA-SN charts at all shifts. We noticed that performance of the RCUSUM-SR scheme also increased as the value of *m* increased as compared to the other schemes under study (cf. Fig 2). At the end, it is we may conclude that the RCUSUM-SR chart is more useful in detecting all the selected shifts under all the considered distributions.

## 4. Application of the proposed chart

Application of the proposed chart is presented in this section. The data set is supplied by Muttlak and Al-Sabah [5] and collected in Pepsi Cola Company in Al-Khobar, Saudi Arabia.

From Muttlak and Al-Sabah [5] data set using RSS technique, 27 ranked set samples were chosen by repeating cycle 4 times and every cycle has size *n* = 3, so *r* = 12 and during the filling process it was observed that internal pressure system of machines got high that led to the increment in the quantity of soft drink bottles. Management indicated the quality control manager that the problem occurred and observed at the 19^th^ sample point of data collection. The proposed scheme is compared with REWMA-SR scheme. We also chosen 27 samples having size *r* = 12 using SRS scheme for comparing the RCUSUM-SR chart to the CUSUM-SR, EWMA-SR and EWMA-SN charts. We have chosen the design constants of the proposed scheme and other schemes by setting up *ARL*_0_ ≅ 370 for the construction of the control limits and these values are given in Table 8.

**Table 8:** Values of design parameters and control limits for the proposed and existing control charts.

Charts	(*K*,*H*)	(*λ*,*L*)	*LCL*	*CL*	*UCL*
RCUSUM – SR	(2, 11.45)	−	−	−	−
CUSUM − SR	(1, 2.3)	−	−	−	−
REWMA – SR	−	(0.05, 2.118)	-6.395	0.000	6.395
EWMA − SR	−	(0.05, 2.487)	-10.153	0.000	10.153
EWMA − SN	−	(0.05, 2.118)	-1.381	0.000	1.381

**Fig 3:**
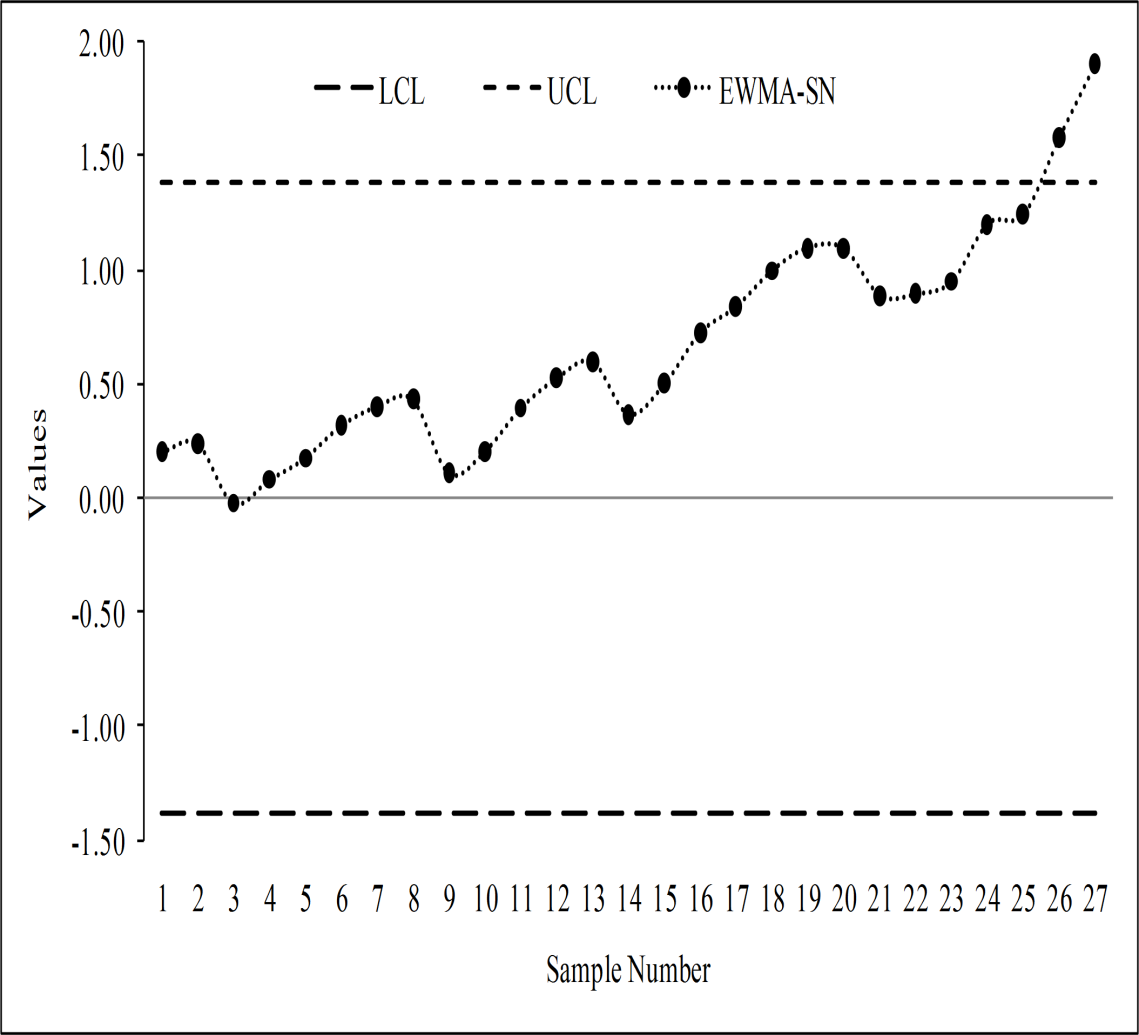
The EWMA-SN chart for the real data set.

**Fig 4:**
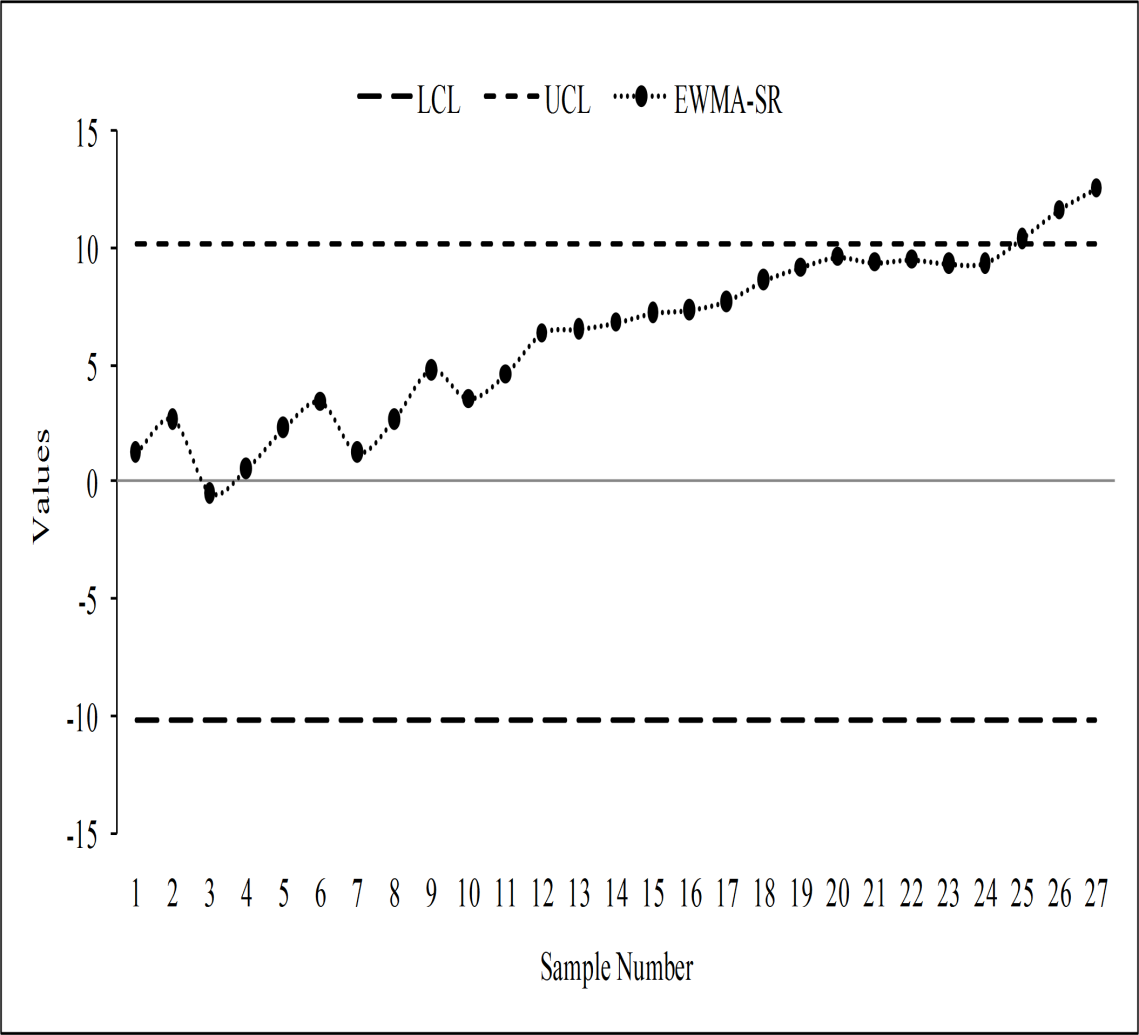
The EWMA-SR chart for the real data set.

**Fig 5:**
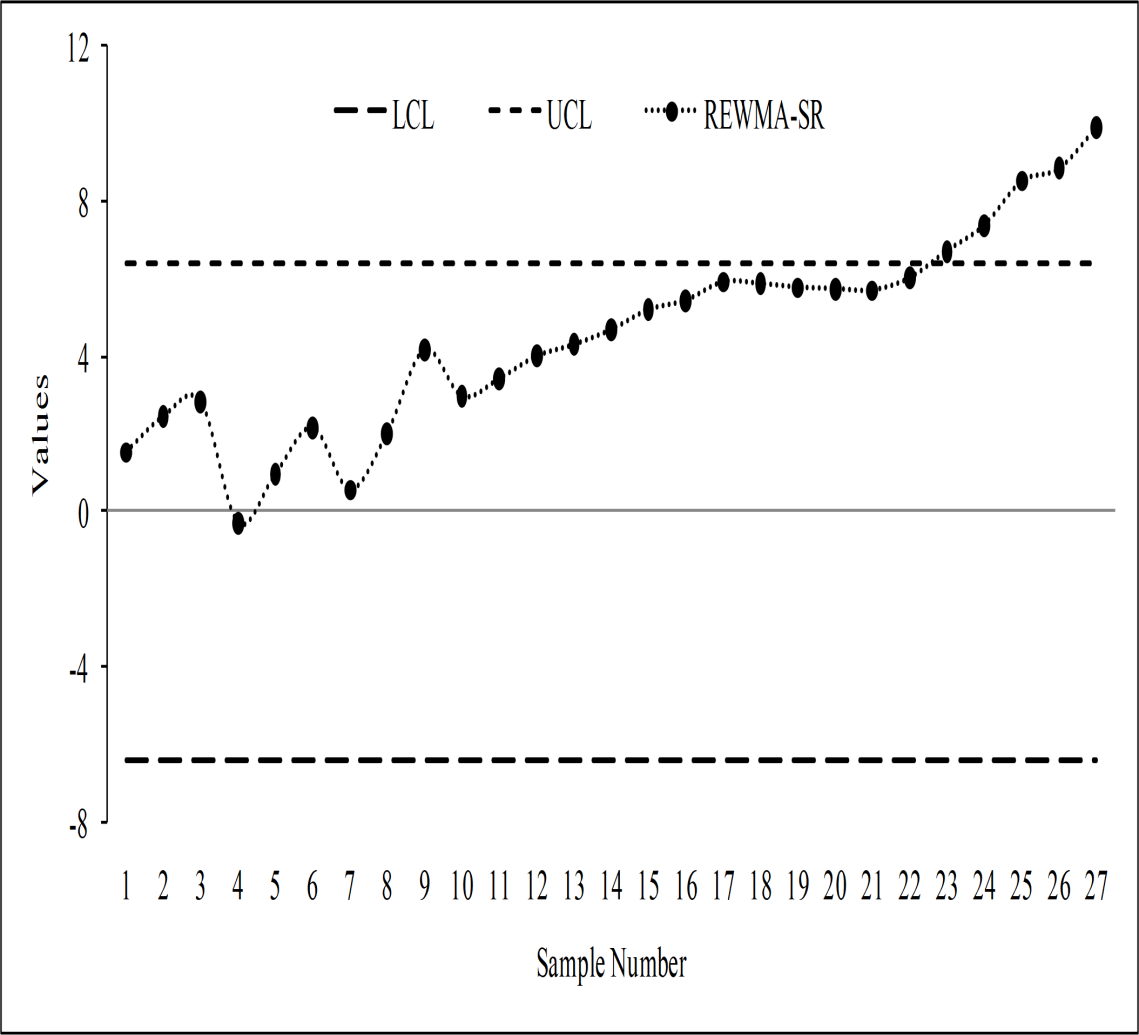
The REWMA-SR chart for the real data set.

**Fig 6:**
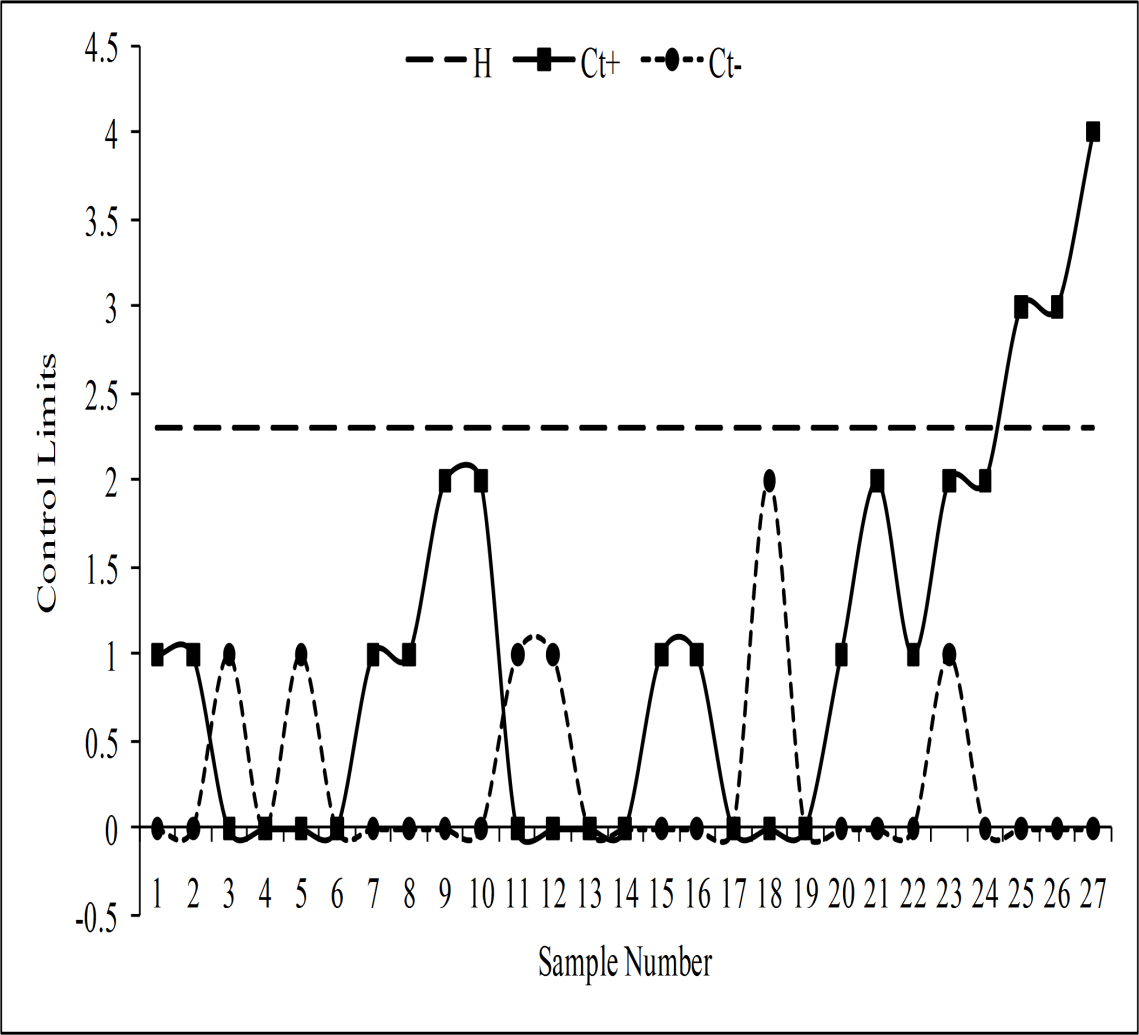
The CUSUM-SR chart for the real data set.

**Fig 7:**
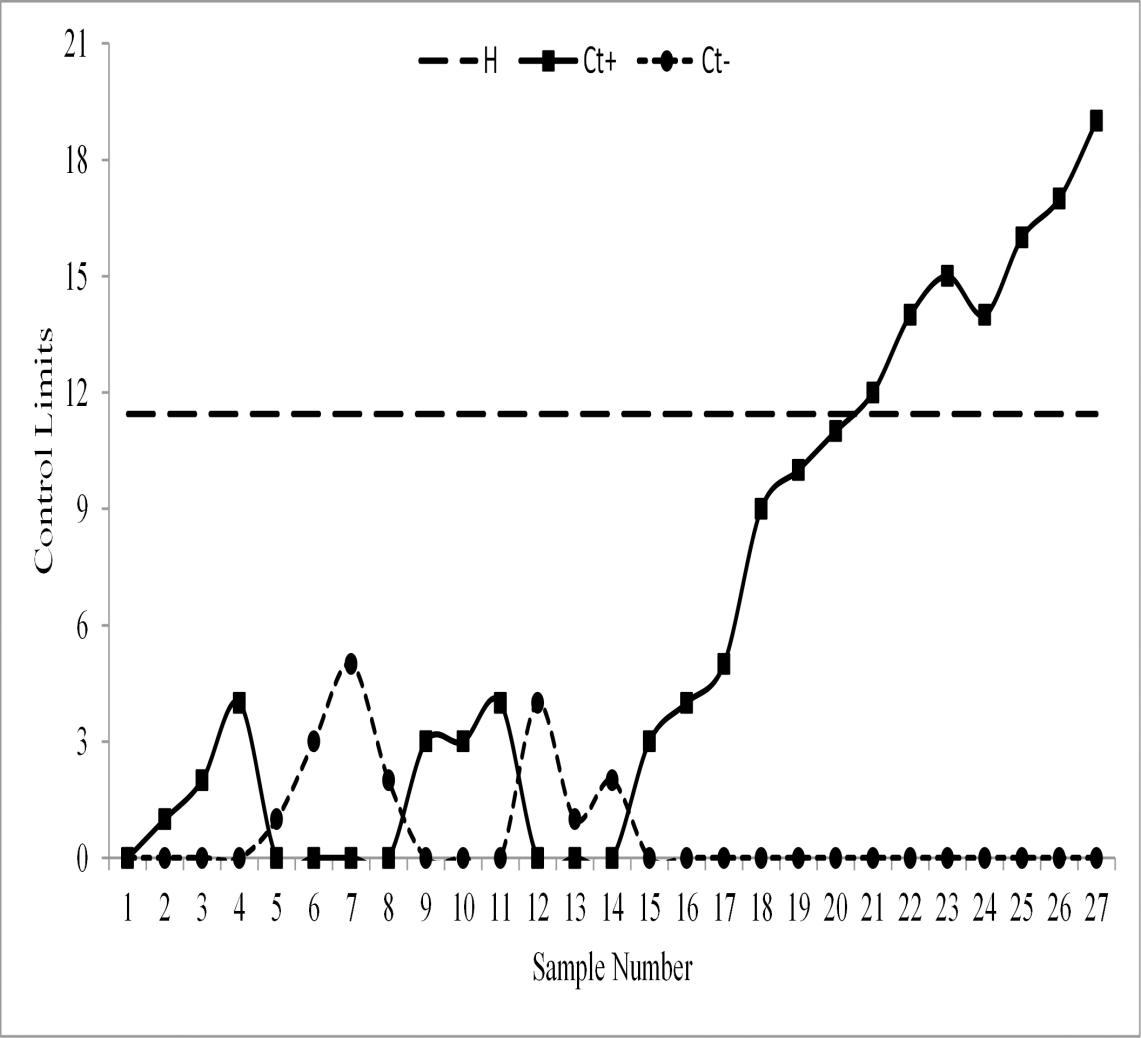
The proposed chart for the real data set.

The monitoring statistics of the EWMA-SN, the EWMA-SR, the REWMA-SR, the CUSUM-SR and the RCUSUM-SR schemes are thus plotted against their control limits as depicted in Figs 3, 4, 5, 6 and 7 respectively. We observed that the EWMA-SN scheme triggered two out of control warning sign at samples 26 and 27 (cf. Fig 3). On the other hand, the EWMA-SR and the CUSUM-SR charts proposed by Bakir and Reynolds [18] and Graham et al. [25], respectively, indicated out of control signals at samples 25, 26 and 27 (cf. Figs 4 and 6). The REWMA-SR chart gave out of control signs at samples 23, 24, 25, 26 and 27 (cf. Fig 5). Finally, the RCUSUM-SR chart triggered seven out of control signals at samples 21, 22, 23, 24, 25, 26 and 27 (cf. Fig 7). So, we can conclude that the proposed scheme has a quicker shift detection capability as compared to the EWMA-SN, the EWMA-SR, the REWMA-SR and the CUSUM-SR schemes.

## 5. Conclusions and recommendations

Control charts are extensively used to monitor stability and performance of processes with an objective of detecting abnormal variations in process parameters. In this paper, we have applied ranked set sampling scheme in order to propose a non-parametric CUSUM control chart based on Wilcoxon signed rank test statistic to monitor the process location. The proposed chart is compared with the REWMA-SR, the CUSUM-SR chart, the EWMA-SN and the EWMA-SR charts. From the results of this study, we have observed that the proposed scheme shows a quicker detection ability as compared to its competitors. Hence, it is concluded that when the units are difficult and costly to measure, the proposed non-parametric CUSUM Wilcoxon signed rank control chart, based on ranked set sampling may be preferred to its existing counterparts. It is to be mentioned that the scope of this work can also be extended to other control charting strategies and diversities of runs rules schemes.

## Supporting information

S1 Dataset (XLSX)
